# The effects of aquatic and land-based interventions on children with developmental coordination disorder

**DOI:** 10.3389/fnhum.2025.1638987

**Published:** 2025-11-13

**Authors:** Lúcio Fernandes Ferreira, Cleverton José Farias de Souza, Jorge Alberto de Oliveira, Andrea Michele Freudenheim

**Affiliations:** 1 Laboratory for Studies in Human Motor Behavior, Faculty of Physical Education and Physiotherapy, Federal University of Amazonas, Manaus, Brazil; 2 Motor Behavior Laboratory, School of Physical Education and Sport, University of São Paulo, São Paulo, Brazil

**Keywords:** Amazonian, child development, motor performance, motor skills disorders, effectiveness of treatment, terrestrial intervention, liquid environment

## Abstract

**Background:**

Knowledge about the effects of aquatic intervention on children with developmental coordination disorder (DCD), as well as the maintenance of these effects over time, is still scarce. We believe that there is a need to advance our knowledge of this subject, for which we have set ourselves the following objectives: (1) to test whether the effects of aquatic and land-based interventions influence the motor performance of children with DCD; (2) to check whether these effects persist over time; and (3) whether the effects of aquatic and land-based interventions bring motor performance values closer together between children with DCD and those with typical development.

**Methods:**

76 children aged between 6 and 10 years old were divided into four groups, control developmental coordination disorder (C-DCD); control typical development (C-TD); experimental terrestrial developmental coordination disorder (T-DCD); and experimental aquatic developmental coordination disorder (A-DCD). Due to some dropouts, the final sample consisted of 66 children, 27 girls and 39 boys. The groups of children with DCD were randomized according to their initial total score on the Movement Assessment Battery for Children Test - second edition (MABC-2). The analysis of variance (one-way ANOVA) carried out in the pre-test showed similarity between the means of the DCD groups (C-DCD, T-DCD and A-DCD), which ensured that these groups started from the same performance level. The interventions lasted 4.5 months (18 weeks) with three sessions a week, totaling 54 sessions of 60 min each. The experimental groups took part in the same intervention protocol, with the only difference being the environment (A-DCD = aquatic environment; T-DCD = terrestrial environment).

**Results:**

Analysis of the results revealed no significant effect for the group and time point interaction, *F*(6, 133) = 1.36, *p* = 0.235. However, effects were detected for group, *F*(2, 54.7) = 3.78, *p* < 0.05, and for time point *F*(3, 131) = 22.91, *p* < 0.001. The Tukey-Kramer *post-hoc* test found a difference between the T-DCD and C-DCD groups (*p* < 0.05; independent-groups effect size (d_*ig*_ = 0.85; d_*ig*_ = 0.87; d_*ig*_ = 0.92). For time point, differences were found between the pre-test and the other time points [post-intervention (repeated measures effect size) (d_*R,M*_ = 1.14), 3 months post-test (d_*R,M*_ = 1.51) and 6 months post-test (d_*R,M*_ = 2.2)] with a significance level of *p* < 0.001. For the A-DCD group there was no statistically significant difference in relation to either the C-DCD or T-DCD group, but we did observe large effect size values [pre-test and post-test (d_*R,M*_ = 1.14), pre-test and 3 months-post-test (d_*R,M*_ = 1.29) and pre-test and 6 months-post-test (d_*R,M*_ = 1.61)]. Regarding the analysis of the Z score, the results indicated an interaction effect for the group and time point *F*(6, 132) = 2.30, *p* = 0.038. This difference was located by *post hoc* between the C-DCD group and the A-DCD (*p* < 0.05; independent groups pre-test - post-test effect size (_*digpp*_ = 1.72) and _*dig*_ = 0.79) and T-DCD (*p* < 0.05 ES _*digpp*_ = 1.65 and _*dig*_ = 0.79) groups at 6 months-post-test.

**Conclusion:**

We conclude that both aquatic and terrestrial interventions have positive effects on the motor performance of children with DCD, that these effects are maintained over time but also bring the motor performance of children with DCD closer to that of children with typical development.

## 1 Introduction

Developmental coordination disorder (DCD) is characterized by marked impairments in the development of motor coordination, which affects the performance of manipulation, locomotion and balance skills, in a general or specific way. It significantly affects a child’s participation in activities of daily living (ADL) and school (ADS) (Smits-Engelsman et al., 2025; Tamplain et al., 2024; Farhat et al., 2024; Landgren et al., 2021; Magalhães et al., 2011; Miller et al., 2021; Sugden et al., 2008; Kirby and Sugden, 2007; Zoia et al., 2006; Sugden et al., 2006). It is a complex condition of as yet unknown cause, whose diagnosis is based on symptoms that are recurrent and independent of culture, race and socioeconomic status (Gao et al., 2024; Blank et al., 2019; Smits-Engeslman et al., 2012; Lingam et al., 2009; Kadesjo and Gillberg, 1999).

Its incidence in the school-age child population is 6% (American Psychiatric Association, 2013), which already makes it one of the most frequent disorders in childhood (Zwicker et al., 2012; Wann, 2007), and the majority of children identified with DCD continue to present motor disorders in adolescence and adulthood (McQuillan et al., 2021; Poulsen et al., 2008; Sugden et al., 2008; Zoia et al., 2006; Sugden et al., 2006; Cantell et al., 2003).

Developmental coordination disorder has been a subject of interest to researchers who aim to understand its cause, improve diagnosis and investigate the effects of intervention programs (Gao et al., 2024; Ayyash and Preece, 2003). Studies examining the effects of intervention programs on DCD have looked at a variety of aspects, such as the influence of different developmental disorders (comorbidities) on the results of the programs (Watemberg et al., 2007); the response of DCD subgroups to interventions (Green et al., 2008); comparison between individual application and group application (Hung and Pang, 2010); the effects of graded exergames on fitness performance (Smits-Engelsman et al., 2021), and comparison between teacher-guided and parent-guided programs (Sugden and Chambers, 2003; Rintala et al., 1998).

Moreover, examining the efficacy of different perspectives of motor interventions for children, Smits-Engeslman et al. (2012) concluded that interventions approaches from a task-oriented perspective yields stronger effects than process-oriented approaches and Ghorbanzadeth et al. (2024) that the combination of the teaching games for understanding (TGFU) and sport education (SE) teaching method is the best method for improving the motor proficiency of DCD children compared to teaching games for understanding TGFU, SE, and linear methods.

In general, the results of these studies have shown that intervention programs have the potential to improve the motor performance of children with DCD, reducing primary impairments (e.g., disorders with motor skills related to manipulation, stabilization, and locomotion) and possibly secondary impairments (e.g., negative consequences on self-esteem, self-concept, perception of competence, and social relationship). However, while in Polatajko et al. (1995) and Eliasson et al. (2003) the effects on short-term motor performance were not resistant to time, in two other studies (Sugden and Chambers, 2003, 2006) they remained in the post intervention long term tests. In this sense the results concerning the maintenance of these effects over time are still controversial.

Nonetheless, with the exception of one exploratory study, the studies that have investigated the effects of intervention programs on the DCD population have only used the terrestrial environment (clinics, school gyms and the home) and little is known about the maintenance of these effects on motor performance after the intervention has ended, i.e., over time. On the other hand, the aquatic environment has been the most used environment in studies that have investigated the effects of intervention programs for people with atypical development (cerebral palsy, autistic and visually impaired) and its effects have been studied frequently (Ogonowska-Slodownik et al., 2024; Zhao et al., 2024; Vodakova et al., 2022; Dimitrijevic et al., 2012; Getz et al., 2012; Jorgic et al., 2012; Pan, 2011, 2010; Fragala-Pinkham et al., 2008).

A study conducted by Jorgic et al. (2012), for example, evaluated seven children with cerebral palsy, aged between 8 and 10 years, who only underwent aquatic intervention over a period of 6 weeks. The results indicated significant improvements in the performance of walking, running and jumping skills. Another study just investigating the effects of aquatic intervention on the motor performance of children with cerebral palsy was carried out by Dimitrijevic et al. (2012). The participants were 27 children aged between 5 and 14 years old, divided into two groups – control and experimental. The intervention was applied for 6 weeks. The results showed that the motor performance of the experimental group, i.e., the one that underwent aquatic intervention, was superior to that of the control group. However, the effects obtained as a result of the intervention were not maintained over time.

The researchers argued that the effects of aquatic intervention are enhanced by the specific properties of this environment. The viscosity combined with the hydrostatic pressure favors muscle strengthening without damaging the tissues or causing too much tension on specific parts of the body. They also state that the hydrostatic pressure exerted on blood vessels causes an increase in respiratory and circulatory activity and an increase in muscle tone, making motor performance more efficient (Getz et al., 2012; Fragala-Pinkham et al., 2008). Also noteworthy is the important role of buoyancy in supporting the joints and stabilizing the body. The more a body is immersed, the greater the action of buoyancy and, consequently, the lower the overload exerted by the action of gravity (Getz et al., 2006, 2012; Pan, 2011).

By analogy, we understand that the aquatic environment also has the potential to enhance the effects of interventions on the motor performance of children with DCD. However, we found only one study that investigated the effects of aquatic intervention on this population. Hillier et al. (2010) selected 12 children with DCD between the ages of 5 and 8 years and divided them into two groups – control (non-intervention) and experimental. Intervention was applied for 6 weeks, with one session per week, and addressed specific tasks of static balance, ball skills and walking/running.

No significant differences were found from pre-test to post-test. These results indicate that the aquatic intervention program had no significant effects on children with DCD. However, these results should be interpreted with caution as the intragroup analysis revealed that the experimental group decreased by 20% (3.9 points) their total score on the MABC test, indicating that motor performance improved after the intervention, while the control group increased by 14% (2.6 points), indicating impairments in motor performance. According to the researchers, although no significant differences were observed between the groups, those results lend clinical significance to the aquatic intervention program for the motor performance of children with DCD.

It is also important to consider the reduced number of weekly sessions (1) and the short period of application of the intervention (6 weeks), as intervention studies carried out in the terrestrial environment indicate that the weekly frequency combined with longer periods of application of the program are important variables (Polatajko et al., 1995; Eliasson et al., 2003; Sugden and Chambers, 2003).

Considering the information above, we would like to emphasize that knowledge about the effects of aquatic intervention on children with DCD, as well as about the maintenance of these effects over time, is still scarce. As for land-based intervention, all of the studies show positive effects on motor performance. However, the results concerning the maintenance of these effects over time are still controversial. We therefore believe that there is a need to advance our knowledge of this subject, for which we have set ourselves the following research questions: (1) Are there effects of aquatic and land-based interventions on the motor performance of children with DCD? (2) Do these effects persist over time? and (3) Do the effects of aquatic and land-based interventions bring motor performance values closer together between children with DCD and those with typical development? Hereto, the following objectives were pursued in this study: (1) to test whether the effects of aquatic and land-based interventions influence the motor performance of children with DCD; (2) to check whether these effects persist over time; and (3) to investigate whether the effects of aquatic and land-based interventions are capable of narrowing the gap in motor performance between children with DCD and those with typical development.

## 2 Materials and methods

### 2.1 Participants

This study was conducted with 1.200 children, aged 6 – 10 years who were attending three public schools in the South-central region of Manaus, Brazil. The children in this study had predominantly brown features, which guarantees the homogeneity of the sample regarding socioeconomic status and ethnic characteristics. As this is a study with a school sample, we emphasize that this age group was specifically chosen due to the fact that in Manaus, children begin their academic activities by the age of six with the start of their first year of primary school, since the provision of early childhood education is still very scarce. Moreover, we consider that it is optimal to identify and intervene as soon as possible when it comes to motor difficulties.

Thus, out of the total, 153 children were pre-selected by teachers in the classroom based on evidence of motor difficulties with a negative impact on daily life activities – such as dressing, feeding, riding a bicycle – and/or on academic achievement such as poor handwriting skills, difficulties with gross and/or fine motor skills, locomotion, agility, manual dexterity, complex skills and/or balance. The pre-selected children were submitted to the following inclusion and exclusion criteria: (a) motor skill deficit; (b) activities of daily and school living impact; (c) early onset symptoms begin in the early developmental period; and (d) rule out other conditions (e.g., cerebral palsy, hemiplegia, or muscular dystrophy); or a pervasive developmental disorder, according to DSM-V (American Psychiatric Association, 2013).

Meeting the criteria for the selection of the sample, 52 children were then classified into three groups of DCD, namely, A-DCD (aquatic intervention); T-DCD (terrestrial intervention); and C-DCD (control DCD), that did not participate in the intervention. 35 children with typical development were selected for the control typical development group (C-TD), of which 24 met the following inclusion criteria: (a) total MABC-2 score ≥ 25th percentile. The exclusion criteria adopted for the C-TD group were: (a) learning difficulties; and (b) social difficulties.

After that, and due to some dropouts, 66 children, aged between 6 and 10 years old, 27 girls and 39 boys [7.6 ± 1.0 years and months (age), 25.00 ± 4.92 kg (body mass), 1.24 ± 0.76 m (height)] were divided into four groups: two controls (C-DCD and C-TD) and two experimental (T-DCD and A-DCD) (Table 1).

**TABLE 1:** Relative frequency (%).

Gender				Measures		
Groups	Female	Male	Total	Age (years)	Weight (kg)	Height (cm)
				Mean ± SD	Mean ± SD	Mean ± SD
A-DCD	6 (32%)	13 (68%)	19 (100%)	7.4 ± 0.17	24.85 ± 1.13	1.24 ± 0.02
T-DCD	4 (25%)	12 (75%)	16 (100%)	7.6 ± 0.27	25.13 ± 1.03	1.24 ± 0.01
C-DCD	8 (67%)	4 (33%)	12 (100%)	7.3 ± 0.32	24.36 ± 0.88	1.24 ± 0.18
C-TD	9 (48%)	10 (52%)	19 (100%)	7.9 ± 0.23	25.44 ± 1.49	1.25 ± 0.02
*P*-value				0.05	0.05	0.05

Means and standard deviations (±SD) for age, weight, and height of the experimental groups (A-DCD and T-DCD) and controls (C-DCD and C-TD). One-way ANOVA analysis did not detect any significant differences in age, weight, or height (*p* > 0.05).

The children with DCD were randomly divided into three groups. The analysis of variance (one-way ANOVA) carried out in the pre-test showed similarity between the means of the C-DCD, T-DCD and A-DCD groups [*F*(2) = 0.592, *p* = 0.55] which ensured that these groups started from the same performance level. Table 2 shows the composition of the groups in terms of the severity of the motor disorder.

**TABLE 2:** Composition of the groups regarding the severity condition of the motor disorder.

	Severity condition of the DCD	
Groups	Severe DCD (≥5° percentil)	Moderate DCD (≤6° and ≥16° percentiles)
A-DCD	11 (57%)	8 (43%)
T-DCD	8 (50%)	8 (50%)
C-DCD	6 (50%)	6 (50%)

Absolute and relative (%) frequencies of the children with severe and moderate developmental coordination disorder (DCD). The one-way ANOVA ensured that the groups started from the same performance level [*F*(2) = 0.592, *p* = 0.55].

Regarding socio-economic status, we found that the majority of families of children with DCD (82.4%) had a monthly income of less than three times the minimum wage and 67.7% of the families were made up of more than five people. As for the parents’ level of education, the majority had completed/incomplete elementary school (44.1%) followed by completed/incomplete higher education (38.2%). As for their parents’ profession, 64.7% worked as freelancers (informal), traders or employees in the industrial district.

From the physiotherapeutic assessment, we found that most of the children in the sample with characteristics of DCD were born at term, and only one was born after the gestation period. Weight and height were in the normal range (Table 3).

**TABLE 3:** Absolute and relative (%) frequencies of cases, mean, and ± standard deviation (SD) of factors related to the birth of children with developmental coordination disorder (DCD).

Gestation period							
Groups				Weight (kg)		Height (cm)	
	Full-term	Post-term	Not reported	Mean	± SD	Mean	± SD
A-DCD	13 (68.4%)	1 (5.3%)	5 (26.3%)	3.249	0.543	0.46	0.08
T-DCD	12 (75%)	0 (0%)	4 (25%)	3.080	0.612	0.49	0.01
C-DCD	4 (33%)	0 (0%)	8 (67%)	3.215	0.200	0.48	0.01

In addition, two children in the T-DCD group had complications (unspecified) at birth, and another two children started speaking between the ages of 4 and 5. Of the A-DCD group, three had complications at birth (use of forceps; hypoxia; broken/dislocated collarbone). Regarding possible associated difficulties, the physiotherapy assessment identified the following: (a) T-DCD group: learning difficulties, reading difficulties, writing difficulties, language difficulties, hyperactivity and lack of concentration; (b) A-DCD group: learning difficulties, language difficulties, hyperactivity and lack of concentration; (c) C-DCD group: learning difficulties, language difficulties, lack of concentration.

### 2.2 Study design

**FIGURE 1:**
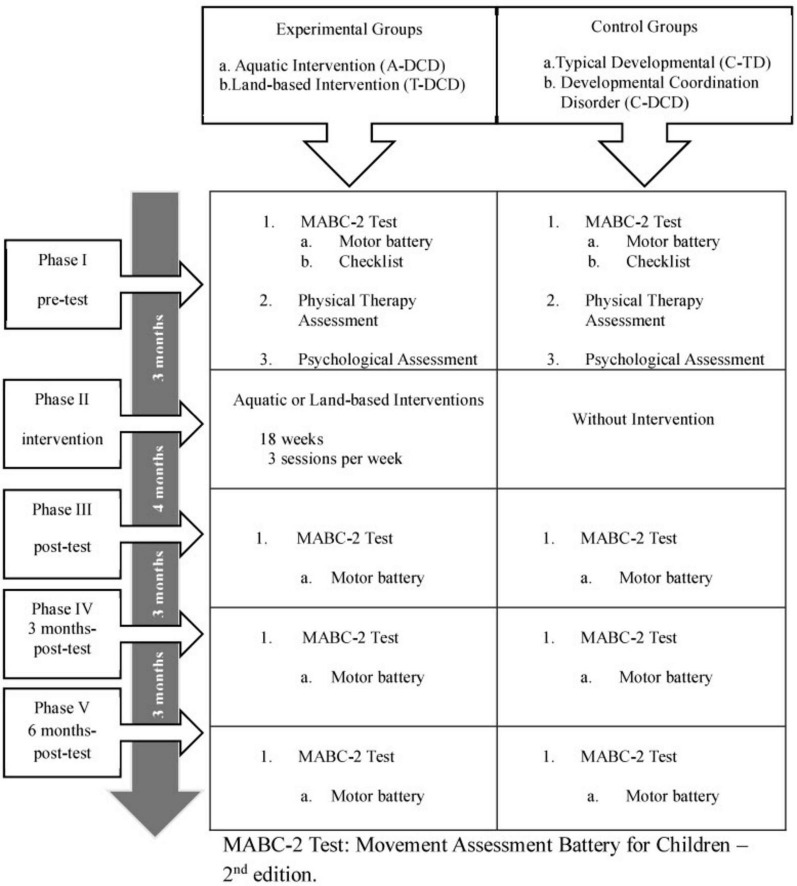
Study design.

In the pre-test, the motor battery (MABC-2) was used to measure the motor performance of the four groups. Next, the experimental groups underwent interventions (aquatic or terrestrial) for a period of 4.5 months with a frequency of three weekly classes of 60 min each, totaling 54 sessions. The children in the control groups continued with their normal activities. At the end of the interventions, we reapplied the MABC-2 test. After a further 3 and 6 months, we carried out the 3-months post-test and 6 months-post-test assessments, respectively (Figure 1).

### 2.3 Tests and assessments

The following assessments were carried out: (a) physiotherapy, in which two physiotherapists (professional identification register cards in Brazil, numbers 99137-F 12 and 96882-F 12) took turns conducting the assessments at the Physiotherapy laboratory of the Nilton Lins University (UniNiltonLins) – Manaus, Amazonas – in the presence of the parents or guardians. The assessments were scheduled in advance and lasted an average of 1 h; (b) intelligence quotient (IQ) assessment, carried out by the Applied Psychology Service (SEPA) of the UniNiltonLins under the responsibility and guidance of the chief psychologist (professional identification register card in Brazil, number 20/5257). The instrument used was the Wechsler Intelligence Scale for Children – WISC-III (Wechsler, 1991), which was applied individually, in a specific room, where only the child and the administrator were present. The assessments were scheduled in advance and lasted an average of 2 h.

This study was approved by the Research Ethics Committees of the School of Physical Education and Sport of the University of São Paulo and UniNiltonLins, under protocol numbers 2011/30 and 043/11, respectively.

#### 2.3.1 The movement assessment battery for children – second edition

The Movement Assessment Battery for Children – Second Edition (MABC-2 Test) (Henderson et al., 2007) identifies children with motor development disorders or delays. This test is useful in clinical and educational contexts and is of great value to researchers in various areas related to child development (Blank et al., 2019), covering the ages of 3–16 years.

The test consists of two instruments: (1) a motor battery and (2) a motor behavior observation checklist. These instruments measure the child in different contexts: while the first prioritizes the experimental context, the second focuses on the everyday context. These instruments complement each other in terms of pre-selecting and identifying children with DCD (Sugden and Wright, 1998) and meet the A and B criteria established by the American Psychiatric Association (2013).

The MABC-2 motor battery is a standardized, norm-referenced test consisting of three sections: (1) manual dexterity; (2) aiming and receiving; and (3) balance. Its tasks are specific to each age group: (range 1) 3–6 years; (range 2) 7–10 years; and (range 3) 11–16 years (Henderson et al., 2007). The sum of the scores for each section results in the child’s total score.

#### 2.3.2 Motor behavior observation checklist

The MABC-2 Test checklist is a revised and reduced version of the original, published in 1992. Its aim is to identify children who may have motor disorders. It was drawn up based on a list of specific motor behaviors, which can be observed in a child’s daily life – at school (forming letters using a pencil or pen and manipulating small objects), in the playground (using fixed equipment/toys and walking/running while avoiding colliding with people), or at home (buttoning their shirt, cutting with scissors and receiving a ball). The child’s performance on each task on the list is scored by an observer in terms of “how competently the task was performed.” These individual values added together provide a total raw score, which is transformed into a percentile.

Ramalho et al. (2013) translated, adapted and verified the presentation, content and construct validity and reliability of the Portuguese version of the MABC2 test checklist. The results indicated high agreement values for relevance and inter-rater agreement (above 90%), appropriate convergent and discriminant validity, high reliability indices (α = 0.94) and inter-rater agreement (ICC between 0.78 and 0.91). Thus, the Portuguese version of the MABC-2 Test checklist proved to be valid and reliable for the Brazilian context.

### 2.4 Intervention protocol

The interventions lasted 4.5 months (18 weeks) with three sessions a week, totaling 54 sessions of 60 min each. The adoption of an extensive practice was based on the previous studies, mainly those of Sugden and Chambers (2003, 2006). The groups took part in the same intervention protocol, with the only difference being the environment (A-DCD = aquatic environment; T-DCD = terrestrial environment).

The approach adopted was task-oriented, thus meeting the recommendation stipulated for planning the intervention (Blank et al., 2019; Smits-Engeslman et al., 2012; Sugden et al., 2006; Pless and Carlsson, 2000). The general plan was based on developmental physical education (Gallahue and Donnelly, 2008) and covered three themes: (1) Stabilization Skills – long jump, jumping from different heights, jumping hurdles, balancing on two legs, balancing on one leg, balancing on a crossbar and balancing on different surfaces; (2) Locomotion Skills – walking, running, sliding, galloping, hopping on the same leg and hopping on alternate legs; and (3) Manipulative Skills – receiving with two hands, throwing overarm, throwing underarm, volleying and rebounding. One central theme was developed each week, and two motor skills from this central theme were practiced. The order of the themes, the motor skills practiced, and the materials used were the same, so that the only factor to differ was the medium, and this was intentional, as this was the central aspect to the questions in this study. It should also be noted that the aquatic intervention was not concerned with teaching swimming skills, an aspect that is very present in studies involving the aquatic environment.

The lessons were distributed as follows: 2 weeks of adaptation (6 lessons), 5 weeks for stabilization skills (15 lessons), 5 weeks for locomotion skills (15 lessons) and 5 weeks for manipulation skills (15 lessons). The first 2 weeks were used to adapt the children to their specific intervention environment, class timetable, materials and equipment. Each week a theme was developed and two motor skills related to this theme were practiced, one on each day of the class, with the two motor skills being repeated in the third class of the week.

In order to ensure that the adherence rate, stipulated as 70% of a total of 54 sessions, was reached and that the subjects’ data could be taken into account in the statistical analysis, transportation was made available for the children.

### 2.5 Dependent variables (operational variables)

1) Total score of the MABC-2 Test (raw score) was obtained from the sum of the sub-scores of each of the subsections that make up the test (manual dexterity, aiming and receiving, balance). It corresponds to the motor performance of children with DCD. It was used to verify the effects of the interventions (aquatic and terrestrial).

2) Z score (standardized score) was calculated by subtracting the individual raw score from the mean divided by the standard deviation for the C-TD group’s pre-test values. It indicates how far above or below average the motor performance of children with DCD is in terms of standardized deviation units. Its purpose was to check how close the motor performance of children with DCD was to that of the C-TD group. It is important to mention that no significant differences were found between the four measures (pre-test, post-test, 3 months-post-test, and 6 months-post-test) of the C-TD group, which is why the pre-test values were chosen.

### 2.6 Statistical analysis

The normality of the data and the absence of extreme observations (outliers) were guaranteed using the Shapiro-Wilks test and by standard visual inspection, confirming the necessary assumptions for parametric analysis.

For the inferential analyses involving the total score of the MABC-2 Test and the total score of the MABC-2 Test transformed into a Z score as a function of the initial values of the C-TD group, we used mixed models with group (C-DCD; T-DCD; A-DCD) and time (pre-test; post-test; 3 months-post-test; and 6 months-post-test) as fixed factors and individuals as a random factor (Ugrinowitsch et al., 2004). The Kenward-Roger adjustment was used to deal with the effect of unbalanced groups on the degrees of freedom. For cases of significant *F*-values, Tukey-Kramer adjustments were used for multiple comparisons. The significance level adopted was *p* ≤ 0.05.

Effect size calculations were carried out according to Morris and DeShon (2002) and Becker (1988). The classification of values was based on Cohen’s proposal [as cited in Nakagawa and Cuthill (2007)]: small (*d* = 0.2), medium (*d* = 0.5) and large (*d* = 0.8).

## 3 Results

### 3.1 The MABC-2 total score

Analysis of the results revealed no significant effect for the group and time interaction, *F*(6, 133) = 1.36, *p* = 0.235. However, main effects were detected for group, *F*(2, 54.7) = 3.78, *p* < 0.05, and time point *F*(3, 131) = 22.91, *p* < 0.001. The Tukey-Kramer *post-hoc* test found a difference between the T-DCD and C-DCD groups (*p* < 0.05; ES d_*ig*_ = 0.85; d_*ig*_ = 0.87; d_*ig*_ = 0.92). For the time factor, differences were found between the pre-test and the other time points [post-test (ES: d_*R,M*_ = 1.14), 3 months-post-test (ES: d_*R,M*_ = 1.51) and 6 months-post-test (ES: d_*R,M*_ = 2.2)] with a significance level of *p* < 0.001.

**FIGURE 2:**
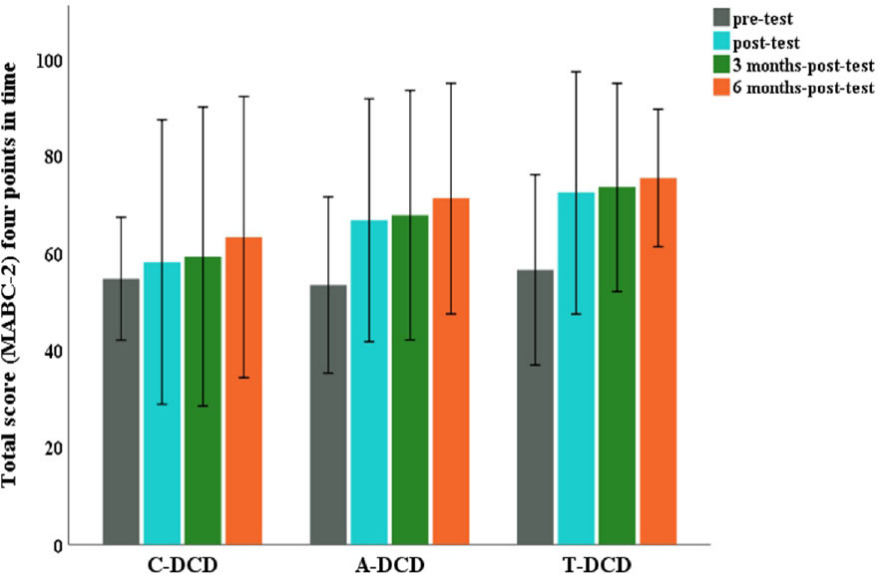
Total scores reflecting the motor performance of the experimental groups (A-DCD and T-DCD) and the control group (C-DCD) across time points (pre-test, post-test, 3 months-post-test, and 6 months-post-test).

For the A-DCD group there was no statistically significant difference in relation to either the C-DCD or T-DCD group, but we did observe large effect size values [pre-test and post (ES: d_*R,M*_ = 1.14), pre-test and 3 months-post-test (ES: d_*R,M*_ = 1.29) and pre-test and 6 months-post-test (ES: d_*R,M*_ = 1.61)] (Figure 2).

### 3.2 Z score

The results indicated an interaction effect for the group and time factors *F*(6, 132) = 2.30, *p* = 0.038. This difference was located by *post hoc* between the C-DCD group and the A-DCD (*p* < 0.05 ES _digpp_ = 1.72 and _dig_ = 0.79) and T-DCD (*p* < 0.05 ES _digpp_ = 1.65 and _dig_ = 0.79) groups at 6 months-post-test.

**FIGURE 3:**
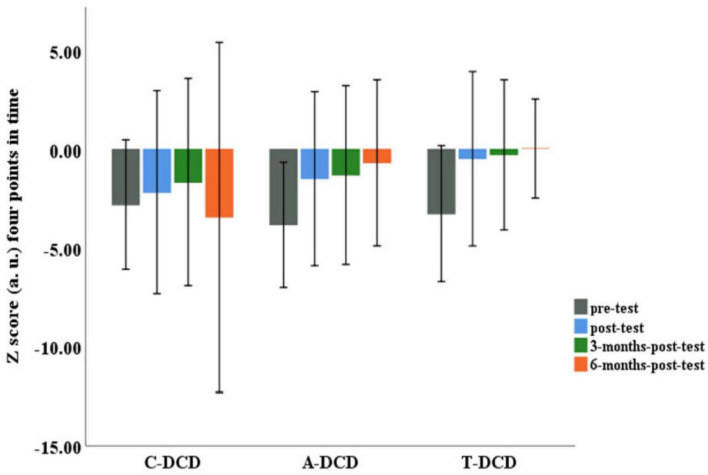
Z-scores (arbitrary units) indicating the distance between the performances of the control group (C-DCD) and experimental groups (A-DCD and T-DCD) relative to the performance of the typically developing control group (C-TD) across time points (pre-test, post-test, 3 months-post-test, and 6 months-post-test). There was a significant difference between the experimental groups and the control group (*p* < 0.05).

For the A-DCD group, the Tukey-Kramer test found significant differences between pre-test and post-test [ES d_R_,_M_ = 1.22), pre-test and 3 months-post-test (ES d_R_,_M_ = 1.29) and pre-test and 6 months-post-test (ES d_R_,_M_) = 1.69] with a significance level of *p* < 0.05. For the T-DCD group, there were also differences between pre-test and post-test (ES d_R_,_M_ = 1.97), pre-test and 3 months-post-test (d_R_,_M_ = 1.66) and pre-test and 6 months-post-test (d_R_,_M_ = 2.19) with a significance level of *p* < 0.05. Unlike the experimental groups, the C-DCD group showed no significant differences between pre-test and post-test (ES d_R_,_M_ = 0.44), pre-test and 3 months-post-test (d_R_,_M_ = 0.32) and pre-test and 6 months-post-test (d_R_,_M_ = 0.07) (Figure 3).

## 4 Discussion

The research questions posed were: (1) Are there effects of aquatic and land-based interventions on the motor performance of children with DCD? (2) Do these effects persist over time? and (3) Do the effects of aquatic and land-based interventions bring motor performance values closer together between children with DCD and those with typical development?

With regard to the first question, the results allowed us to answer in the affirmative, i.e., that the terrestrial and aquatic interventions had a positive influence on the motor performance of children with DCD.

Results involving the motor performance scores do not indicate a significant difference between the T-DCD and A-DCD groups but revealed between the T-DCD and C-DCD groups. T-DCD group showed significant results immediately after the end of the intervention. As the three DCD groups (A-DCD, T-DCD and C-DCD) started from the same performance level, it can be inferred that the difference found between the T-DCD and C-DCD groups was due to the land-based intervention. For the time factor, differences were found between the pre-test and the other times, i.e., post-test, 3 months-post-test and 6 months-post-test. These effects were of great magnitude, suggesting that there was clinical significance for the T-DCD group over time. This result indicates that there was an improvement in motor performance over time, with no distinction between groups.

Taken together, these results reinforce the evidence on the effectiveness of land-based interventions for children with DCD (Green et al., 2008; Niemeijer et al., 2007; Watemberg et al., 2007; Sugden and Chambers, 2003, 2006; Schoemaker et al., 2003; Pless et al., 2000; Kaplan et al., 1993).

On the other hand, the analysis involving the motor performance scores revealed no significant difference between the A-DCD and C-DCD groups. The beneficial effects of the aquatic intervention found for the A-DCD group were not enough to differentiate it statistically from the control group (C-DCD). We suggest that considering the intervention environment, it is possible that the improvement in motor performance in skills performed in the aquatic environment is not completely transferred to the performance of equivalent motor skills in the terrestrial environment, requiring a longer time for this improvement to take effect, as the results here have shown. Transfer, for example, is a subject of research in the area of Motor Learning (Schmidt and Young, 1987; Holding, 1976).

As mentioned, the results of the present study showed that no differences were detected between the T-DCD and A-DCD groups. However, the T-DCD group showed significant results immediately after the end of the intervention and when the magnitude of the effect was analyzed, it showed effects ranging from large to small over the course of the evaluations compared to the A-DCD group. We believe that this result reinforces the argument that the children in the A-DCD group found it difficult to transfer motor skills developed in the aquatic environment to equivalent ones performed on land.

The results of this study are in line with those of Hillier et al. (2010), who also found no significant difference between the experimental and control groups after aquatic intervention, although they did observe an increase in the motor performance score in 50% of the children in the experimental group. In the same way, in our study, with the groups starting from the same level, as evidenced by the magnitude of the effect sizes found, the children in the A-DCD group showed greater improvements in motor performance over time than those in the C-DCD group. This confirms the clinical significance of the aquatic intervention mentioned by Hillier et al. (2010).

Beyond that, maybe because the number of sessions was higher, our results go further than those found by Hillier et al. (2010). In the present study the A-DCD group showed significant intra-group effects in motor performance after the intervention, as well as in the 3- and 6-months post-tests.

Summarizing, this study showed that both land-based and aquatic interventions were effective in improving the motor performance of children with DCD. Moreover, although the results do not indicate a significant difference between the T-DCD and A-DCD groups, we observed a slight superiority of the land-based intervention over the water-based one.

The second question arises because we assume, however, that the effectiveness of an intervention is only confirmed if the effects are maintained over time. This was in fact observed in the present study because differences were found between the pre-test and the other times, i.e., post-test, 3 months-post-test and 6 months-post-test. These results indicate that the effects were maintained over the 6 months following the intervention for both experimental DCD groups.

Studies looking at the maintenance of the effects of interventions are rare (Smits-Engeslman et al., 2012). We believe that our study makes it possible to advance knowledge regarding land-based interventions, since it was found that the effects were maintained in evaluations after the end of the intervention. The design of this study, specifically with regard to the length (4.5 months or 18 weeks) and with three sessions a week complies with the recommendations in the literature, specifically involving the planning of intervention programs (Sugden et al., 2006; Pless and Carlsson, 2000). By comparison, the interventions in the studies by Polatajko et al. (1995) and Eliasson et al. (2003) comprised a reduced number of weeks (5 and 9, and 8, respectively), which we believe may have influenced the failure to maintain the effects achieved with these interventions.

Specifically, concerning aquatic intervention and children with DCD, we found no studies focusing on the maintenance of effects after intervention. In this respect, the results of the present study, showing the effectiveness of aquatic intervention for individuals with DCD (intragroup significant differences), also represent an advance in this field of research.

In relation to the third question of the study, with regard to the Z score, no differences were detected between the T-DCD and A-DCD groups. We therefore consider that the aquatic intervention showed similar benefits to the land-based intervention in terms of its tendency toward the reference for children with typical development.

In turn, the analysis of the Z score showed that the T-DCD group differed from the C-DCD group in terms of its approximation to the reference for children with typical development 6 months after stopping the intervention. It should be noted that these groups started from the same performance level, i.e., before the intervention they both differed equally from the benchmark for children with typical development. The results show that the T-DCD group was closer to the reference. These effects were of great magnitude, attesting to the clinical significance of the terrestrial intervention.

The analysis of the Z score showed that also the A-DCD and C-DCD groups differed in terms of how close they were to the reference for children with typical development 6 months after the intervention. This was not the case with the C-DCD group. It should be noted that these groups started from the same performance level. As with T-DCD, the results show that the A-DCD group was closer to the reference. However, only the A-DCD group showed a trajectory toward the reference group which began shortly after the intervention and became similar to it after 6 months. These results strengthen claims about the potential of the aquatic environment to help children with motor disorders of different etiologies (Zhao et al., 2024; Ogonowska-Slodownik et al., 2024; Pan, 2011; Hillier et al., 2010; Fragala-Pinkham et al., 2008; Getz et al., 2006, 2007); by extension, they have also shown that children with DCD without access to intervention tend to experience maintenance or worsening of their motor disorder (Cantell et al., 1994; Geuze and Borger, 1993; Losse et al., 1991; Gillberg et al., 1989).

Taken together these results suggest that, in relation to the reference, in functional terms, in the same way as for T-DCD, the A-DCD group became distanced from the C-DCD group. In this sense, although the land-based intervention enabled performance similar to that of typically developing children more quickly, the results suggest that the T-DCD and A-DCD groups got similarly close to the typical reference, which indicates similarity in their effectiveness in bringing the motor performance of children with DCD closer to that of children with typical development.

The results presented here serve as a warning, as they reinforce that without intervention, the difference between the developmental trajectory of children with DCD in relation to that of typical children can continue throughout adolescence and into adulthood (Payne and Ward, 2019; Tal-Saban and Kirby, 2018; Tal-Saban et al., 2012, 2014; Missiúna et al., 2007; Taylor et al., 2007; Henderson and Henderson, 2002; Cantell et al., 2001, 2003), negatively interfering with their development and quality of life (Cavalcante-Neto et al., 2025; Khairati et al., 2024; Nobre et al., 2023; Draghi et al., 2021; Zwicker et al., 2018; Liberman et al., 2013; Tal-Saban et al., 2012; Wagner et al., 2012; Zwicker et al., 2012; Bejerot et al., 2011; Hill et al., 2011; Rivilis et al., 2011; Engel-Yeger and Kasis, 2010; Missiúna et al., 2007; Sigmundsson et al., 1998).

Finally, they reinforce the statement by Tal-Saban et al. (2012) that regardless of the severity of the motor disorder, children with DCD need help to bring their developmental trajectory closer to that of children considered to have typical development.

In short, aquatic and terrestrial interventions were similar in terms of their effectiveness for children with DCD, as both equally benefited motor performance. It seems, then, that the environment in which the intervention takes place is not the main factor influencing improvements in children with DCD. In this study, the aquatic and terrestrial interventions were equivalent in terms of duration – 4, 5 months/18 weeks/54 lessons. Following existing recommendations, the weekly frequency was three sessions for both interventions (Sugden and Chambers, 2003, 2006; Pless and Carlsson, 2000). In addition, the thematic content developed in the sessions was equivalent.

Considering these specificities, the results of this study allow us to state that the effectiveness of the aquatic intervention and the land-based intervention are equivalent in terms of the benefits related to the motor performance of children with DCD.

## 5 Conclusion

We conclude that both aquatic and land-based interventions have positive effects on the motor performance of children with DCD and that these effects are not only maintained over time, but also bring the motor performance of children with DCD closer to that of children with typical development. However, we would point out that the land-based intervention proved to be effective immediately after it ended, while the aquatic intervention took longer to prove its effectiveness. In short, motor intervention, regardless of the medium in which it takes place, aquatic or terrestrial, is effective in treating children with DCD.

Based on these results and conclusions, future studies should be carried out with the aim, for example, of investigating the effects of aquatic intervention in relation to the skills specific to each of the subsections of the motor battery of the MABC-2 test. It is possible that aquatic intervention, due to the properties of the environment, particularly favors stabilization skills, for example. Another question that calls for future research concerns the interactions between the subtype of DCD, the severity of the disorder and the environment (aquatic or land-based), with regard to the outcome of the intervention.

## Data Availability

The original contributions presented in this study are included in this article/supplementary material, further inquiries can be directed to the corresponding author.

## References

[B1] American Psychiatric Association. (2013). *DSM-V-TR: diagnostic and statistical manual of mental disorders.* Washington, DC: APA.

[B2] Ayyash H. F. Preece P. M. (2003). Evidence-based treatment of motor coordination disorder. *Curr. Paediatrics* 13 360–364. 10.1016/S0957-5839(03)00058-7

[B3] Becker B. J. (1988). Synthesizing standardized mean-change measures. *Br. J. Math. Stat. Psychol.* 41 257–278. 10.1111/j.2044-8317.1988.tb00901.x

[B4] Bejerot S. Edgar J. Humble M. B. (2011). Poor performance in physical education – a risk factor for bully victimization: A case-control study. *Acta Paediatrics* 100 413–419. 10.1111/j.1651-2227.2010.02016.x 21039827

[B5] Blank R. Barnett A. L. Cairney J. Green D. Kirby A. Polatajko H. (2019). International clinical practice recommendations on the definition, diagnosis, assessment, intervention, and psychosocial aspects of developmental coordination disorder. *Dev. Med. Child Neurol.* 61 242–285. 10.1111/dmcn.14132 30671947 PMC6850610

[B6] Cantell M. H. Kooistra L. Larkin D. (2001). Approaches to intervention in children with developmental coordination disorder. *N. Z. J. Disabil. Stud.* 9 106–119. 10.1123/apaq.11.2.115

[B7] Cantell M. H. Smyth M. M. Ahonem T. P. (1994). Clumsiness in adolescence: Educational, motor, and social outcomes of motor delay detected at 5 years. *Adapted Phys. Activity Quart.* 11 115–129. 10.1123/apaq.11.2.115

[B8] Cantell M. H. Smyth M. M. Ahonen T. P. (2003). Two distinct pathways for developmental coordination disorder: Persistence and resolution. *Hum. Mov. Sci.* 22 413–431. 10.1016/j.humov.2003.0.002 14624826

[B9] Cavalcante-Neto J. L. Brito R. S. Silva L. S. O. Zamunér A. R. (2025). Assessment of cardiac outcomes of children and adolescents with autism spectrum disorder and developmental coordination disorder: A systematic review. *Res. Autism* 125:202604. 10.1016/j.reia.2025.202604

[B10] Dimitrijevic L. Alexsandrovic M. Madic D. Okicic T. Radovanovic D. Daly D. (2012). The effects of aquatic intervention on the gross motor function and aquatic skills in children with cerebral palsy. *J. Hum. Kinetics* 32 167–174. 10.2478/v10078-012-0033-5 23487257 PMC3590865

[B11] Draghi T. T. G. Cavalcante Neto J. L. Tudela E. (2021). Symptoms of anxiety and depression in schoolchildren with and without developmental coordination disorder. *J. Health Psychol.* 26 1519–1527. 10.1177/13591053198725 31556324

[B12] Eliasson A. C. Rösblad B. Häger-Ross C. (2003). Control of reaching movements in 6 year-old prematurely born children with motor problems – An intervention study. *Adv. Physiotherapy* 5 33–48. 10.1080/14038190310005780

[B13] Engel-Yeger B. Kasis A. H. (2010). The relationship between developmental co-ordination disorders, child’s perceived self-efficacy and preference to participate in daily activities. *Child Care Health Dev.* 36 670–677. 10.1111/j.1365-2214.2010.01073.x 20412146

[B14] Farhat F. Denysschen M. Mezghani N. Kammoun M. M. Gharbi A. Rebai H. (2024). Activities of daily living, self-efficacy and motor skill related fitness and the interrelation in children with moderate and severe developmental coordination disorder. *PLoS One* 19:e0299646. 10.1371/journal.plne.0299646 38652708 PMC11037543

[B15] Fragala-Pinkham M. Haley S. M. O’neil M. E. (2008). Group aquatic aerobic exercise for children with disabilities. *Dev. Med. Child Neurol.* 50 822–827. 10.1111/j.1469-8749.2008.03086.x 19046177

[B16] Gallahue D. L. Donnelly F. C. (2008). *Developmental Physical Education for all children.* Phorte: São Paulo.

[B17] Gao J. Song W. Zhong Y. Huang D. Wang J. Zhang A. (2024). Children with developmental coordination disorders: A review of approaches to assessment and intervention. *Front. Neurol.* 15:1359955. 10.3389/fneur.2024.1359955 38846037 PMC11153681

[B18] Getz M. Hutzler Y. Vermeer A. (2006). Effects of aquatic interventions in children with neuromotor impairments: A systematic review of the literature. *Clin. Rehabil.* 20 927–937. 10.1177/0269215506070693 17065536

[B19] Getz M. Hutzler Y. Vermeer A. (2007). The effects of aquatic intervention on perceived physical competence and social acceptance in children with cerebral palsy. *Eur. J. Special Needs Educ.* 22 217–228. 10.1080/08856250701269705

[B20] Getz M. Hutzler Y. Vermeer A. Yarom Y. Unnithan V. (2012). The effect of aquatic and land-based training on the metabolic cost of walking and motor performance in children with cerebral palsy: A pilot study. *Int. Scholarly Res. Netw.* 12:8. 10.5402/2012/657979

[B21] Geuze R. H. Borger H. (1993). Children who are clumsy: Five years late. *Adapted Phys. Activity Quart.* 10 10–21. 10.1123/apaq.10.1.10

[B22] Ghorbanzadeth B. Kirazci S. Badicu G. (2024). Comparison of the effects of teaching games for understanding, sport education, combined and linear pedagogy on motor proficiency of children with developmental coordination disorder. *Front. Psychol.* 15:1385289. 10.3389/fpsyg.2024.1385289. 38863663 PMC11166209

[B23] Gillberg I. C. Gillberg C. Groth J. (1989). Children with preschool minor neurodevelopmental disorders V: Neurodevelopmental profiles at age 13. *Dev. Med. Child Neurol.* 31 14–24. 10.1111/j.1469-8749.1989.tb08407.x 2920868

[B24] Green D. Chambers M. Sugden D. A. (2008). Does subtype of developmental coordination disorder count: Is there a differential effect on outcome following intervention? *Hum. Mov. Sci.* 27 363–382. 10.1016/j.humov.2008.02.009 18400322

[B25] Henderson S. E. Henderson L. (2002). Toward an understanding of developmental coordination disorder. *Adapted Phys. Activity Quart.* 19 12–31. 10.1123/apaq.19.1.11 28195798

[B26] Henderson S. E. Sugden D. A. Barnett A. L. (2007). *Movement assessment battery for children (examiner’s manual)*, 2nd Edn. London: Harcourt Assessment.

[B27] Hill E. L. Brown D. Sorgardt K. S. (2011). A preliminary investigation of quality-of-life satisfaction reports in emerging adults with and without developmental coordination disorder. *J. Adult Dev.* 18 130–134. 10.1007/s10804-011-9122-2

[B28] Hillier S. Mcintyre A. Plummer L. (2010). Aquatic physical therapy for children with developmental coordination disorder: A pilot randomized controlled trial. *Phys. Occup. Therapy Pediatr.* 30 111–124. 10.3109/01942630903543575 20367516

[B29] Holding D. H. (1976). An approximate transfer surface. *J. Motor Behav.* 8 1–9. 10.1080/00222895.1976.10735049 23952791

[B30] Hung W. W. Y. Pang M. Y. C. (2010). Effects of group-based versus individual-based exercise training on motor performance in children with developmental coordination disorder: A randomized controlled pilot study. *J. Rehabil. Med.* 42 122–128. 10.2340/16501977-0496 20140407

[B31] Jorgic B. Dimitrijevic L. Aleksandrovic M. Okicic T. Madic D. Radovanovic D. (2012). The swimming program effects on the gross motor function, mental adjustment to the aquatic environment, and swimming skills in children with cerebral palsy: A pilot study. *Specijalna Edukacija I Rehabilitacija* 11 51–66. 10.5937/specedreh1201051j

[B32] Kadesjo B. Gillberg C. (1999). Developmental coordination disorder in Swedish 7-year-old children. *J. Am. Acad. Child Adolesc. Psychiatry* 38 820–828. 10.1097/00004583-199907000-00011 10405499

[B33] Kaplan B. J. Polatajko H. J. Wilson B. N. Faris P. D. (1993). Reexamination of sensory integration treatment: A combination of two efficacy studies. *J. Learn. Disabil.* 26 342–347. 10.1177/002221949302600507 8492053

[B34] Khairati F. Stewart N. Zwicker J. G. (2024). How developmental coordination disorder affects daily life. *Adolesc. Perspect.* 144:104640. 10.1016/j.ridd.2023.104640 38056031

[B35] Kirby A. Sugden D. A. (2007). Children with developmental coordination disorders. *J. R. Soc. Med.* 100 182–186. 10.1177/014107680710011414 17404341 PMC1847727

[B36] Landgren V. Fernell E. Gillberg C. Landgren M. Johnson M. (2021). Attention-deficit/hyperactivity disorder with developmental coordination disorder: 24-year follow-up of a population-based sample. *BMC Psychiatry* 21:161. 10.1186/s12888-021-03154-w 33752617 PMC7983399

[B37] Liberman L. Ratzon N. Bart O. (2013). The profile of performance skills and emotional factors in the context of participation among young children with developmental coordination disorder. *Res. Dev. Disabil.* 34 87–94. 10.1016/j.ridd.2012.07.019 22940162

[B38] Lingam R. Hunt L. Golding J. Jongmans M. Emond A. (2009). A. prevalence of developmental coordination disorder using The DSM IV at 7 years of age: A UK population based study. *Pediatrics* 123 693–700. 10.1542/peds.2008-1770 19336359

[B39] Losse A. Henderson S. E. Elliman D. Hall D. Knight E. Jongmans M. (1991). Clumsiness in children-do they grow out of it? a 10-year follow-up study. *Dev. Med. Child Neurol.* 33 55–68. 10.1111/j.1469-8749.1991.tb14785.x 1704864

[B40] Magalhães L. C. Cardoso A. A. Missiúna C. (2011). Activities and participation in children with developmental coordination disorder: A systematic review. *Res. Dev. Coordination* 32 1309–1316. 10.1016/j.ridd.2011.01029 21330100

[B41] McQuillan V. A. Swanwick R. A. Chambers M. E. Schlüter D. K. Sugden D. A. (2021). A comparison of characteristics, develomental disorders and motor progression between children with and without developmental coordination disorder. *Hum. Mov. Sci.* 78:102823. 10.1016/j.humov.2021.102823 34051667

[B42] Miller H. L. Sherrod G. M. Mauk J. E. Fears N. E. Hynan L. S. Tamplain P. M. (2021). Shared features or Co-occurrence? Evaluating symptoms of developmental coordination disorder in children and adolescents with Autism spectrum disorder. *J. Autism Dev. Disord.* 51 3443–3455. 10.1007/s10803-020-04766-z 33387238 PMC10177628

[B43] Missiúna C. Moll S. King S. King G. Law M. (2007). A trajectory of troubles: Parents’ impressions of the impact of developmental coordination disorder. *Phys. Occup. Therapy Pediatr.* 27 81–98. 10.1080/j006v27n01_06 17298942

[B44] Morris S. B. DeShon R. P. (2002). Combining effect size estimates in meta-analysis with repeated measures and independent-groups designs. *Pshychol. Methods* 7 105–125. 10.1037/1082-989X.7.1.105 11928886

[B45] Nakagawa S. Cuthill I. C. (2007). Effect size, confidence interval and statistical significance: A practical guide for biologists. *Biol. Rev.* 82 591–605. 10.1111/j.1469-185X.2007.00027.x 17944619

[B46] Niemeijer A. S. Smits-Engelsman B. C. Schoemaker M. M. (2007). Neuromotor task training for children with developmental coordination disorder: A controlled trial. *Dev. Med. Child Neurol.* 49 406–411. 10.1111/j.1469-8749.2007.00406.x 17518923

[B47] Nobre G. C. Ramalho M. H. S. Ribas M. S. Valentini N. C. (2023). Motor, physical, and psychosocial parameters of children with and without developmental coordination disorder: A comparative and associative study. *Int. J. Environ. Res. Public Health* 20:2801. 10.3390/ijerph200442801 36833496 PMC9956583

[B48] Ogonowska-Slodownik A. Jakobowicz O. Alexander L. Marinho-Buzelli A. R. Morgulec-Adamowicz N. (2024). Aquatic therapy in children and adolescentes with disabilities: A scoping review. *Children* 11:1404. 10.390/children11111404 39594979 PMC11593235

[B49] Pan C. Y. (2010). Effects of water exercise swimming program on aquatic skills and social behaviors in children with autism spectrum disorders. *J. Autism Dev. Disord.* 14 9–28. 10.1177/1362361309339496 20124502

[B50] Pan C. Y. (2011). The efficacy of an aquatic program on physical fitness and aquatic skills in children with and without autism spectrum disorders. *Res. Autism Spectrum Disord.* 5 657–665. 10.1016/j.rasd.2010.08.001

[B51] Payne S. Ward G. (2019). Conceptual framework of developmental coordination disorder in adolescence: Findings from a qualitative study. *Br. J. Occup. Therapy* 83 246–255. 10.1177/0308022619867620

[B52] Pless M. Carlsson M. (2000). Effects of motor skill intervention on developmental coordination disorder: A meta-analysis. *Adapted Phys. Activity Quart.* 17 381–401. 10.1123/apaq.17.4.381

[B53] Pless M. Carlsson M. Sundelin C. Persson K. (2000). Effects of group motor skill intervention on five-to-six-year-old children with developmental coordination disorder. *Pediatric Phys. Therapy* 12 183–189. 17091030

[B54] Polatajko H. J. Macnab J. J. Anstett B. Malloy-Miller T. Murphy K. Noh S. (1995). Clinical trial of the process-oriented treatment approach for children with developmental coordination disorder. *Dev. Med. Child Neurol.* 37 310–319. 10.1111/j.1469-8749.1995.tb12009.x 7535267

[B55] Poulsen A. A. Ziviani J. M. Johnson H. Cuskelly M. (2008). Loneliness and life satisfaction of boys with development coordination disorder: The impact of leisure participation and perceived freedom in leisure. *Hum. Mov. Sci.* 27 325–343. 10.1016/j.humov.2008.02.004 18353475

[B56] Ramalho M. H. S. Valentini N. C. Muraro C. F. Gadens R. Nobre G. C. (2013). Validação da língua portuguesa: lista de checagem da movement assessment battery for children. *Motriz* 19 423–431. 10.1590/S1980-65742013000200019

[B57] Rintala P. Pienimäki K. Ahonen T. Cantell M. Kooistra L. (1998). The effects of a psychomotor training program on motor skill developmental language disorders. *Hum. Mov. Sci.* 17 721–737. 10.1016/S0167-9457(98)00021-9

[B58] Rivilis I. Hay J. Cairney J. Klentrou P. Liu J. Faught B. E. (2011). Physical activity and fitness in children with developmental coordination disorder: A systematic review. *Res. Dev. Disabil.* 32 894–910. 10.1016/j.ridd.2011.01.017 21310588

[B59] Schmidt R. A. Young D. E. (1987). “Transfer of movement control in motor learning,” in *Transfer of learning (00. 47-79)*, eds Cormier S. M. Hangman J. D. (Orlando, FL: Academic Press).

[B60] Schoemaker M. M. Niemeijer A. S. Reynders K. Smits-Engelsman B. C. M. (2003). Effectiveness of neuromotor task training for children with developmental coordination disorder: A pilot study. *Neural Plasticity* 10 155–163. 10.1155/NP.2003.155 14640316 PMC2565416

[B61] Sigmundsson H. Pedersen A. V. Whiting H. T. Ingvaldsen R. P. (1998). We can cure child’s clumsiness! A review of intervention methods. *Scand. J. Rehabil. Med.* 30 101–106. 10.1080/003655098444200 9606772

[B62] Smits-Engelsman B. C. M. Denysschen M. Lust J. Coetzee D. Valtr L. Schoemaker M. (2025). Which outcomes are key to the pre-intervention assessment profile of a child with developmental coordination disorder: A systematic review and meta-analysis. *Biomed. J.* 48:100768. 10.1016/j.bj.2024.100768 39032866 PMC12020856

[B63] Smits-Engelsman B. Bonney E. Ferguson G. (2021). Effects of graded exergames on fitness performance in elementary school children with developmental coordination disorder. *Front. Sports Active Living* 3:653851. 10.3389/fspor.2021.653851 33969297 PMC8100245

[B64] Smits-Engeslman B. C. M. Blank R. Van Der Kaay A. C. Van der Meijs R. M. Vlugt-Van Den, Brand E. (2012). Efficacy of interventions to improve motor performance in children with developmental coordination disorder: A combined systematic review and meta-analysis. *Dev. Med. Child Neurol.* 55 229–237. 10.1111/dmcn.12008 23106530

[B65] Sugden D. Chambers M. Utley A. (2006). *Leeds consensus statement 2006: Developmental coordination disorder as a specific learning difficult. ESRC Research Seminar Series 2004–2005*. Leeds: Dyscovery Centre.

[B66] Sugden D. A. Chambers M. E. (2003). Intervention in children with developmental coordination disorder: The role of parents and teachers. *Br. J. Educ. Psychol.* 73 545–561. 10.1348/000709903322591235 14713377

[B67] Sugden D. A. Chambers M. E. (2006). Stability and change in children with developmental coordination disorder. *Child Care Health Dev.* 33 520–528. 10.1111/j.1365-2214.2006.00707.x 17725773

[B68] Sugden D. A. Wright H. C. (1998). *Motor coordination disorders in children.* Thousand Oaks, CA: Sage.

[B69] Sugden D. Chambers M. Utley A. (2006). *Leeds consensus statement.* Leeds.

[B70] Sugden D. Kirby A. Dunford C. (2008). Issues surrounding children with developmental coordination disorder. *Int. J. Disabil. Dev. Educ.* 55 173–187. 10.1080/10349120802033691

[B71] Tal-Saban M. Kirby A. (2018). Adulthood in developmental coordination disorder (DCD): A review of current literature based on ICF perspective. *Curr. Dev. Disord. Rep.* 5 9–17. 10.1007/s40474-018-0126-5

[B72] Tal-Saban M. Ornoy A. Parush S. (2014). Executive function and attention in young adults with and without developmental coordination disorder – A comparative study. *Res. Dev. Disabil.* 35 2644–2650. 10.1016/j.ridd.2014.07.002 25058794

[B73] Tal-Saban M. Zorka S. Grotto I. Ornoy A. Parush S. (2012). The functional profile of young adults with suspected developmental coordination disorder (DCD). *Res. Dev. Disabil.* 33 2193–2202. 10.1016/j.ridd.2012.06.005 22789703

[B74] Tamplain P. Miller H. L. Pavy D. Cermak S. Williams J. Licari M. (2024). The impact for DCD – USA study: The current state of developmental coordination disorder (DCD) in the United States of America. *Res. Dev. Disabil.* 145:104658. 10.1016/j.ridd.2023.104658 38176290 PMC10840388

[B75] Taylor S. Fayed N. Mandich A. (2007). CO-OP intervention for young children with developmental coordination disorder. *Occupation Participation Health* 27 124–130. 10.1177/153944920702700402

[B76] Ugrinowitsch C. Fellingham G. W. Ricard M. D. (2004). Limitations of ordinary least squares models in analyzing repeated measures data. *Med. Sci. Sports Exerc.* 144–2148. 10.1249/01.MSS.0000147580.40591.75 15570152

[B77] Vodakova E. Chatziioannou D. Jesina O. Kudlacek M. (2022). The effect of halliwick method on aquatic skills of children with autism spectrum disorder. *Int J. Environ. Res. Public Health* 19:1620. 10.3390/ijerph192316250 36498324 PMC9738692

[B78] Wagner M. O. Bös K. Jascenoka J. Jekauc D. Petermann F. (2012). Peer problems mediate the relationship between developmental coordination disorder and behavioral problems in school-aged children. *Res. Dev. Disabil.* 33 2072–2079. 10.1016/j.ridd.2012.05.012 22750362

[B79] Wann J. (2007). (Commentary) Current approaches to intervention in children with developmental coordination disorder. *Dev. Med. Child Neurol.* 19 405–405. 10.1111/j.1469-8749.2007.00405.x 17518922

[B80] Watemberg N. Waiserberg N. Zuk L. Lerman-Sagie T. (2007). Developmental coordination disorder in children with attention deficit- hyperactivity disorder and physical therapy intervention. *Dev. Med. Child Neurol.* 49 920–925. 10.1111/j.1469-8749.2007.00920.x 18039239

[B81] Wechsler D. (1991). *WISC-III: Wechsler intelligence scale for children*. São Paulo: Psychologist’s Office.

[B82] Zhao P. Chen K. Zhu G. Li H. Chen S. Hu J. (2024). Effects of aquatic exercise intervention on executive function and brain-derived neurotrophic factor of children with autism spectrum disorder. *Res. Dev. Disabil.* 150:104759. 10.1016/j.ridd.2024.104759 38795553

[B83] Zoia S. Barnett A. Wilson P. Hill E. (2006). Developmental coordination disorder: Current issues. *Child Care Health Dev.* 32 613–618. 10.1111/j.1365-2214.2006.00697.x 17018038

[B84] Zwicker J. G. Harris S. R. Klassen A. F. (2012). Quality of life domains affected in children with developmental coordination disorder: A systematic review. *Child Care Health Dev.* 39 562–580. 10.1111/j.1365-2214.2012.01379.x 22515477

[B85] Zwicker J. G. Suto M. Harris S. R. Vlasakova N. (2018). Developmental coordination disorder is more than a motor problem: Children describe the impact of daily struggles on their quality of life. *Br. J. Occup. Therapy* 1 65–73. 10.1177/0308022617735046

